# Spousal Separation and Use of and Unmet Need for Contraception in Nepal: Results Based on a 2016 Survey

**DOI:** 10.1155/2020/8978041

**Published:** 2020-03-23

**Authors:** Suresh Mehata, Yuba Raj Paudel, Amit Dhungel, Mohan Paudel, Janak Thapa, Deepak Kumar Karki

**Affiliations:** ^1^Ministry of Health and Population, Ram Shah Path, Kathmandu, Nepal; ^2^School of Public Health, University of Alberta, Edmonton, Canada; ^3^United Nations Population Fund (UNFPA) Nepal, Kathmandu, Nepal; ^4^Initiatives for Research Education and Community Health, Kathmandu, Nepal; ^5^Nepal Public Health Research and Development Centre, Kathmandu, Nepal; ^6^Nepal Health Economics Association (NHEA), Kathmandu, Nepal

## Abstract

Nepal is facing a large-scale labour migration—both internal and international—driven by economic and employment opportunities. There is sparse literature available at the national level which examines the link between migration and contraceptive use. This study aimed at identifying contraceptive use and the unmet need for family planning (FP) and exploring its correlates among the married women of reproductive age (MWRA) by their husbands' residence status, using data from Nepal Demographic Health Survey 2016–a nationally representative cross-sectional survey. A stratified two-stage cluster sampling in rural and a three-stage sampling in urban areas were used to select the sampling clusters, and data from 11,040 households were analyzed. Reported values were weighted by sample weights to provide national-level estimates. The adjusted odds ratio (aOR) was calculated using multiple logistic regressions using complex survey design, considering clusters, and stratification by ecological zones. All analyses were performed using Stata 15.0. Among the total MWRA, 53% were using a contraceptive method, whereas the proportion of contraceptive use among the cohabiting couple was 68%. The unmet need for contraceptive use was 10% among cohabiting couples and 50% among the noncohabiting couples. Contraceptive use was significantly low among the women reporting an induced abortion in the last five years and whose husbands were currently away. A strong negative association of spousal separation with contraceptive use was observed (aOR:0.14; *p* < 0.001) after controlling other covariates, whereas a positive association was observed with the unmet need (aOR:8.00; *p* < 0.001). Cohabiting couples had a significantly higher contraceptive use and lower unmet need compared with the couples living apart. Between 2006 and 2016, contraceptive use increased by 1% per year among cohabiting couples, although this increase is hugely attributable to the use of traditional methods, compared with modern methods. The labour migration being a significant and indispensable socioeconomic phenomenon for Nepal, it is necessary to monitor fertility patterns and contraceptive use by cohabitation status in order to ensure that the national family planning interventions are targeted to address the contraceptive and fertility needs of the migrant couples.

## 1. Background

Despite political instability, slow economic growth, and significant geographic and economic barriers to health care, Nepal has made considerable progress in improving reproductive health over the last two decades. Despite these gains, progress on universal access to reproductive health measured by the contraceptive prevalence rate (CPR) and unmet need for family planning remains unsatisfactory. Significant inequalities in use of and unmet need for contraceptives still exist by the place of residence, age-group, and migration status.

In Nepal, international migration and rural-to-urban migration within Nepal [migration may be seasonal, temporary, semipermanent, or permanent; its nature often depends on the reason for migration] are rapidly increasing [1]. A recent study in Nepal showed about a third of the households (30%) had at least one member of the family migrated. The proportion of migration was higher in the rural and disadvantaged regions of Nepal—the mid-western and far-western regions of the country compared with urban and relatively developed regions [2]. The Nepal Household Survey 2012 showed that almost half (47%) of households had at least one member migrating either inside or outside the country [3]. One in ten households (10%) had two migrants, 5% of the households had three migrants, and 7% had four or more migrants. The peak age for male migrants was within the age-groups 20–29 years. Among the male migrants, a majority fell in age-group 15–39 years, with the peak age group of 20–29 years, whereas a majority of female were in 15–29 years age-group [3]. The Nepal Household Survey 2012 also revealed that most of the migrants moved for employment reasons (59%), and this was true among both the group of migrants from urban (53%) and rural (60%) areas. Over a quarter (27%) of households had at least one migrant living overseas, with 6% of the households having at least two migrants and 2% having at least three international migrants (HHS 2012). Rural households (27%) were slightly more likely to have a migrant living overseas than the urban households (24%).

There is sparse literature examining the link between migration and contraceptive use and/or fertility. Using NDHS 2016 data, this study examines spousal separation and its effect on contraceptive use in Nepal [4]. Previous work on this topic using 2011 NDHS data [5] and the data from 40 rural districts [1] showed a significantly higher unmet need and lower use of modern contraceptives among couples living separately compared with cohabiting couples. Spousal separation was shown to play a crucial role in the decline of total fertility in the absence of a corresponding rise in the contraceptive uptake in Nepal [5], which is also supported by a recent mixed method study [2] that identified recently married couples delaying to plan for a baby due to spousal separation, usually husband. In this regard, regular monitoring of contraceptive use according to the changing migration trends and measuring the disaggregated fertility pattern/contraceptive use according to the cohabitation status can help policy makers and planners make an informed decision for family planning programming. This study was conducted to investigate a recent pattern in family planning use according to husband's residence status and study factors associated with the contraceptive use and unmet need for FP among cohabiting and noncohabiting women, to expand the evidence base and help policy makers make evidence based decisions.

## 2. Materials and Methods

Data for this study were drawn from Nepal Demographic Health Survey 2016 [4], a nationally representative survey conducted to provide up-to-date estimates on basic health and demographic indicators. A detailed methodology has been presented in the NDHS 2016 full report [4]. In brief, a stratified two-staged cluster sampling in rural and a three-staged sampling in urban areas were used in order to select the clusters. A total of 383 primary sampling units (PSUs) were selected using probability proportional to their size methods [6]. Subsequently, 30 households per cluster were selected with an equal probability systematic selection criterion. Interviews were completed for 11,040 households, yielding a response rate of 99%.

### 2.1. Variables

The dependent variable in this analysis was use of and unmet need for contraceptives among MWRA currently residing with husbands (cohabiting) and women whose husbands lived away (noncohabiting). Three major predictor variables were included in this analysis: individual factors (such as age, education, had an induced abortion in the last five years, and ever used emergency contraceptives), household-level variables (such as caste/ethnic group and wealth quintile), and community-level variables (such as urban/rural residence, provinces, and ecological zone).The method used to compute the wealth index is described in the NDHS 2016 report [3].

### 2.2. Statistical Analysis

All analyses were performed using Stata version 15 software. Reported values were weighted by sample weights to provide national estimates. Adjusted odds ratio (aOR) was calculated using multiple logistic regression, with all covariates (age, education, ecological zone, place of residence, provinces, wealth quintile, number of living children, caste/ethnicity, women who had induced abortion, and ever used emergency contraceptives) included simultaneously in the model using complex survey design, considering clusters, and stratification by ecological zones. *p* < 0.05 was considered to be statistically significant in this study. Because disaggregating family planning use and the unmet need for family planning by husband's residence status give a more realistic picture of family planning program performance at the population level, we present disaggregated results for the married women of reproductive age (MWRA) currently living with their husbands and women whose husbands lived away [1]. A sensitivity analysis was also carried for noncohabiting couples to identify the duration of separation (≤1 year vs > 1 year) that has more impact on use of and unmet need for family planning.

## 3. Results

Among married women of reproductive age (MWRA), spousal separation was observed among 34% of the participants with a median duration of 11 months (data not shown). Among women whose husbands were currently living away, nearly half of them (49.3%) were separated for more than a year. More than one-third (36%) were separated for less than 6 months, and 14.7% were separated for 6–11 months. Spousal separation was more common among MWRA in rural areas (37%) than their urban counterparts (32%). The peak age for spousal separation was within the age-groups 20–29 years [20–24 (45%) and 25–29 (43%)]. The highest spousal separation was observed among those who reside in Province 4 (42%) and Province 2 (39%), among the third wealth quintile groups (40%) and among those who were Muslims and hill Dalits (39%) (Table 1). Higher spousal separation was observed among women reporting ever use of emergency contraception (39%) than those never had an emergency contraception (34%).

**Figure 1:**
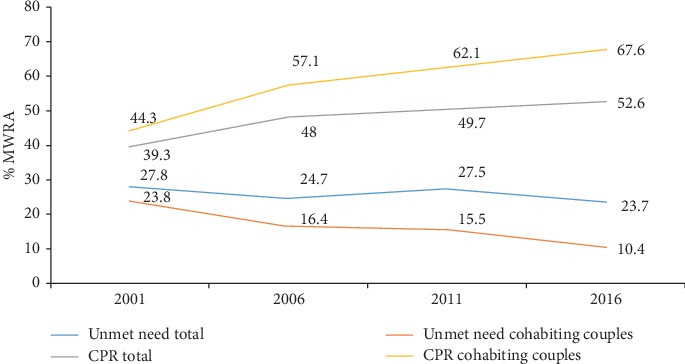
Trend of CPR (any method) and unmet need among total MWRA and the MWRA cohabiting with their husbands (NDHS 2001 to 2016).

Among all MWRA, 53% were using any contraceptives; of whom 81% were using modern methods rather than traditional ones (data not shown). This proportion of contraceptive use (any method) among cohabitating couples increased to 68% (Figure 1), whereas only 24% of women whose husbands lived away reported using any contraceptive methods (Table 2).

The use of contraceptives among women currently residing with husbands and women whose husbands lived away was most common for the age range 35–49 years, the age range when women are most likely to want to stop (rather than delay or space) childbearing. By provinces, among the women currently residing with husbands and women whose husbands lived away, the highest use of contraceptive methods was observed among those residing in Province 7 (74% and 29%, respectively).

The largest gap in contraceptive use (any method) by husband's cohabitation status was observed among those with a higher level of education (55%), those residing in Province 4 (57%) followed by Province 1 (55%), hill Brahmins (59%), and who had and induced abortion in the last five years (60%) (Table 2).

Overall, unmet need of contraceptive use was 24% among any MWRA and 10% among those currently residing with husbands (Figure 1), whereas the unmet need was 50% among those women whose husbands were away. The largest crude difference in unmet need by husband's cohabitation status was observed among couples who had 1–2 living children (45%) compared with other categories (no children, 3–4 children, 5, and more). By age categories, the difference in unmet need by husband's cohabitation was almost similar among age categories covering 15–34 years (39–42%). Those with higher educational status, primary (42%), secondary (47%), and higher education (43%), had large differences in unmet need than those with no education (28%). Province 1 (48%) and Province 4 (50%) had higher difference in unmet need by husband's cohabitation status. Similarly, hills region (45%) had a higher difference in unmet need than the mountain (40%) and Terai regions (35%) of Nepal. By ethnicity, hill Brahmin (48%), hill Chhetri (46%), and hill Janajati (48%) showed the largest crude difference in unmet need than other caste/ethnicities. Women who had an abortion in the last five years had much higher difference in the unmet need for FP (51%) by husbands' cohabitation status than women reporting no abortion in the last five years (39%) (Table 3).

By caste/ethnicity, the lowest use of contraceptive methods was observed among Muslims currently residing with husbands (32%), whereas the highest use was observed among hill Janajati (70%) (Table 2). Among the women whose husbands were away, the less likelihood of using contraceptives was observed among those who had an induced abortion in the last five years (13%) compared with those women who did not have any induced abortion (24%). In the adjusted model, age, number of living children, education, province, caste/ethnicity, and use of induced abortion in the last five years were associated with contraceptive use among those women whose husbands away (Table 4). No difference was found on the impact of the duration of separation (≤1 year vs > 1 year) on use of and unmet need for FP.

In the multivariate model, maternal age, number of living children, education, province, wealth quintile, and caste/ethnicity were associated with contraceptive use among those currently residing with husbands (Table 4). The higher likelihood of contraceptive use was observed among those currently residing with husbands with higher education than the participants with no education (aOR:1.44; 95% CI: 1.02–2.02). Significantly lower contraceptive use was observed among women reporting an induced abortion in the last five years and whose husbands were currently away (aOR:0.42; 95% CI: 0.26–0.68). Reporting of ever use of emergency contraception was not significantly associated with the lower use of contraceptives (any method) both among cohabiting and noncohabiting couples.

Similarly, maternal age, number of living children, province, wealth quintile, caste/ethnicity, and use of induced abortion services in the last five years were associated with unmet need among women currently residing with husbands (Table 4). An inverse relationship of age with the unmet need was observed. Similarly, age, number of living children, education, province, caste/ethnicity, and use of induced abortion services in the last five years were associated with unmet need among women whose husbands were away (Table 4). A strong negative association of spousal separation with contraceptive use was observed (aOR:0.14; *p* < 0.001) after controlling other covariates, whereas a positive association was observed with unmet need (aOR:8.00; *p* < 0.001) (data not shown in table).

## 4. Discussion

Our aim in this study was to study the effect of spousal separation on use of and unmet need for contraceptives in Nepal. The analysis revealed that although family planning use (any method) has slightly increased or leveled off with the any MWRA group, there is an increment of 11% (from 57% in 2006 to 68% in 2016)—with an approximate rise of 1% annually among the cohabiting couples. The overall CPR is 53% among any MWRA, while this rate is much higher at 68% among the cohabiting couples as shown by the NDHS 2016 [4]. Likewise, unmet need for family planning among any MWRA is 24%, whereas this is only 10% among the cohabiting couples. This shows that although overall unmet need remained almost constant between 2006 and 2016, it has decreased by 6% among the cohabiting couples. These findings clearly suggest that presenting aggregate information (without disaggregating the rates with status of cohabitation) on contraceptive use and unmet need can be misinterpreted, which can ultimately affect the appropriate use of scarce resources and prioritization of the family planning program within the neediest population subgroups of the entire MWRA age range. On the other hand, increasing share of the traditional method among the cohabiting couples (4.7% in 2006 to 13.8% in 2016) [4, 5] is another factor to be considered to examine whether this has indeed affected the fertility structure and has not led to increasing unintended pregnancies.

Nepal's labour-related out-migration is male dominated, even though the number of female migrants has seen a rise in recent years. In 2013/2014 alone, of 521,878 total labour migrants, a majority was males (94.5%) [7]. Additionally, a significant number of men and women work in India, who are more likely to be undocumented because of open border policy between India and Nepal [8]. Approximately 8% of Nepalese were out of the country as shown by the 2011 household census, and majority of the out-migrants were of reproductive age [9].

There is a complex and dynamic relationship between migration and fertility. Much of the literature discusses the “fertility-depressing effect” of spousal separation for a short term [10–13]. The fertility depression effect is described as having a reduced fertility rate. Some argue that the fertility-depressing effect is expected to be higher (or more visible) in areas of high fertility and low contraceptive use [13] and also depends on the duration of separation and postpartum amenorrhea [14]. While others argue that migration can result in increased fertility because of higher coital frequency after return from migration [15]. A study from Mozambique revealed that wives of successful migrants were more likely to use modern contraceptives than wives of nonmigrants or less successful migrants [13], thus validating the principle of selective hypothesis [14]. The selective hypothesis proposes that migrants are self-selective groups who consciously control marriage and prefer fewer children [16]. Furthermore, the use of contraceptives when husband is away may depend on service accessibility, sociocultural factors, and women's autonomy. One recent study from Nepal documents that young couples are delaying plan to have a child due to strong socioeconomic pressure [2]. The study explored young couples becoming more conscious to not to bear a child before having themselves economically empowered—which in most cases was to migrate to industrial/developed countries for job opportunities due to increasing unemployment rates at the home country. Increasing aspirations to provide quality education to their kids, the cost of health care, and responsibility to take care of old family members back home has pushed today's youths to migrate out of the country—with Gulf countries becoming the top destination. It appears that migration to countries other than India (which used to be the top destination before) have delayed childbearing because of relatively longer spousal separation. On the other hand, rising general literacy and exposure due to migration must have created an immense pressure on young couples to limit their family size as they become more aware and sensitive about their future and resources needed to plan a family well. With a recent trend of female migration, the study also identified that recently married couples who had unintended pregnancy even opted for abortion, besides economic pressure, which to some extent could have been eased by the legalized and accessible abortion services in Nepal [17]. Furthermore, breakdown of the traditional family structure with a move towards nuclear family might have been another factor behind why couples today plan for a less number of children. With a joint family structure, it is natural that couples have always grandparents and other family members to take responsibility for raising a child. However, recently, this is becoming a less common phenomenon in Nepal. Considering all of these sociocultural factors, we add into the argument that the fertility structure should be seen as a complex and dynamic indicator. Analysis of the impact of spousal separation is the beginning of it, thus moving further from simply concluding the fertility structure as an outcome of a nation's contraceptive prevalence rate.

A large difference in contraceptive use (any method) by husband's cohabitation status among women from well-off groups (higher education and higher wealth quintiles) than those with less education and lower wealth quintile suggests these groups have a higher tendency to refrain from using contraceptives when their husbands are away. Thapa et al. (2014) found that improvements in knowledge and awareness of legalization of abortion, circumstances in which abortion is allowed, and places to obtain services were largely limited among women with higher education and higher wealth quintile [17]. It was estimated that 137,000 legal and over 186,000 illegal abortions were conducted in 2014 in Nepal [18] and figure continue to rise annually [19]. Given the easy availability of surgical and medical abortion in Nepal, educated and wealthy women could have misused abortion service as a means of family planning. Even though the current analysis did not show any association of the ever use of emergency contraceptives with the current use of contraceptives (any method) or unmet need for family planning both among cohabiting and noncohabiting women, over-the-counter availability of emergency contraceptives (ECPs) has proved to be popular among young and educated women in Nepal [20]. ECP use is often under-reported in household surveys such as DHS [20]. Hence, for many noncohabiting women, abortion services and emergency contraceptives could have been the means for meeting fertility regulation needs in Nepal.

This study also found the significantly lower contraceptive use and higher unmet need for family planning among those who had had an induced abortion in the last five years and whose husbands were currently away. A study among women who received surgical abortion in Kathmandu showed that one in five women who had an abortion left the facility without using the family planning method [21]. Almost one-third of the abortion users had a repeat abortion. Regular provision of a range of contraceptives and availability of effective counseling are lacking in many health facilities and clinics in Nepal [21]. Women whose husbands are away experience societal and familial pressure not to use contraceptives. When unplanned pregnancy occurs—either due to husband's unplanned arrival or due to extramarital relationship—abortion becomes the ultimate resort to end unintended pregnancy, which often results in repeated abortions.

Results from multivariate logistic regression showed that age, number of living children, education, province, wealth quintile, and caste/ethnicity were associated with contraceptive use. Increasing age and the higher number of living children were associated with higher chances of using family planning methods. The relative strength of association of increasing age (after 30 years) with the use of modern contraceptives compared with those aged 15–19 years was lower among cohabiting couples than couples living separately. Lindstorm and Giorguli argue that migration has an impact on timing of the first birth and subsequent birth patterns [11]. Cohabiting young couples (15–19 years) may be more likely to delay childbearing by using contraceptives than young couples who separate after marriage. Young couples who live separately might face familial/societal pressure to have children before they separate; however, this needs further investigation.

A significant association was observed between higher levels of education (primary, secondary, and higher) and use of modern methods among cohabiting couples. Given that access to education has increased over time, age-group needs to be taken into account when looking at the relationship between education and use of contraceptives. Additionally, NDHS 2016 found that women with higher education and cohabiting couples were more likely to use traditional methods than those with lower education and couples living separately [4].

### 4.1. Policy Implications

Nepal's case is unique in terms of reduction in total fertility from 4.1 in 2001 to 2.3 in 2016—a reduction of 2 children per women within 15 years' period. The overall status of CPR and unmet need do not fully explain this transition without examining the disaggregated data on contraceptive use and unmet need for family planning by cohabitation status. A further analysis of the NDHS 2011 data revealed that spousal separation played a major role in the reduction of TFR from 3.1 births per women in 2006 to 2.6 per women in 2011 [5].Therefore, it is important to present information on contraceptives use by disaggregating with respect to spousal separation. Furthermore, regular monitoring of family planning indicators according to spousal separation is necessary as the migration trends change. Such a monitoring can help program managers see a real effect of family planning-related interventions and help allocate scarce resources appropriately. Although slightly different from the focus of this study, the growing evidence of the lower birth rate and infertility among migrant couples [22] warrants a major policy reform to manage labour-related migration trend from Nepal to maintain a desired population structure.

### 4.2. Implications for Program

The family planning needs differ significantly between cohabiting and noncohabiting couples. One cannot deny the occurrence of sexual activity among separately living couples [23]. Additionally, spouses need adequate planning in situations where the husband's return is unannounced [1]; therefore, family planning interventions should cater the need of spouse of migrant labours through educational messages such as related to the fertility period, and provision of family planning services. In addition, program should also cater to the needs of specific subgroups such as temporarily separated couples who will be reunited later and couples from different caste/ethnic groups where family planning uptake is very low (Muslims and Terai Dalits) [5].

### 4.3. Future Research Recommendations

We know very little about the family planning needs of migrant population and their spouses. The DHS surveys do not adequately capture fertility desire, unmet need for family planning, and current use of family planning among this population [23]. It is not clear whether spouses of migrants have a lower need for family planning services due to lower coital frequency, whether their complex family planning needs are not being met, or whether emergency contraception or abortion is turning out as a means of family planning. Hence, in-depth qualitative studies are needed to further understand fertility desires, facilitators, and barriers for contraceptive use among spouses of labour migrants in Nepal.

## 5. Conclusion

This study confirms earlier findings by Ban et al. 2012 [1] and Khanal 2013 [5] that spousal separation remains a significant explanatory factor in the use of and unmet need for contraception in Nepal. Among noncohabiting couples, CPR (any method) was at 24%, which is 44 percentage points lower than among the cohabiting couples who had a CPR of 68%. The unmet need for family planning was 50% among noncohabiting couples, while it was 10% among cohabiting couples. This study found that use of family planning methods has increased at a rate of almost 1% per year (between 2006 and 2016) among cohabiting couples, although the overall use of family planning methods seems to have leveled off or slightly increased among total MWRA in Nepal. The labour migration being a significant and indispensable socioeconomic phenomenon for Nepal, it is necessary to monitor fertility patterns and contraceptive use by cohabitation status in order to ensure that the national family planning interventions are targeted to address the contraceptive and fertility needs of the migrant couples.

## Figures and Tables

**Table 1:** Background characteristics of married women by their cohabitation status with their husbands, 2016.

Women's characteristic	Cohabitation status				Total MWRA
	Cohabiting		Noncohabiting		
	*n*	%	*N*	%	
No. of living children					
None	648	63.2	377	36.8	1025
1–2	3066	60.8	1978	39.2	5044
3–4	2148	72.4	817	27.6	2965
5+	659	78.4	181	21.6	840

Age-groups (years)					
15–19	418	59.4	286	40.6	704
20–24	929	55.1	755	44.9	1684
25–29	1118	57.1	839	42.9	1957
30–34	1084	62.8	642	37.2	1726
35–39	1075	71.2	436	28.8	1510
40–44	1030	80.3	253	19.7	1283
45–49	868	85.9	143	14.1	1011

Level of educational					
No education	2784	69.9	1200	30.1	3984
Primary	1182	63.8	670	36.2	1853
Secondary	1797	62.1	1094	37.9	2891
Higher	758	66.1	389	33.9	1147

Place of residence					
Urban	4112	68.2	1919	31.8	6031
Rural	2409	62.7	1434	37.3	3844

Province					
Province 1	1118	67.5	537	32.5	1655
Province 2	1329	61.3	839	38.7	2168
Province 3	1446	75.3	473	24.7	1920
Province 4	553	58.2	397	41.8	950
Province 5	1150	65.8	599	34.2	1749
Province 6	388	66.2	198	33.8	586
Province 7	537	63.5	309	36.5	846

Ecological zone					
Mountain	396	68.7	180	31.3	576
Hill	2791	67.3	1359	32.7	4150
Terai	3334	64.8	1814	35.2	5148

Wealth quintile					
Poorest	1107	65.6	580	34.4	1687
Poorer	1235	63.5	711	36.5	1946
Middle	1263	60.5	825	39.5	2088
Richer	1359	64.5	749	35.5	2107
Richest	1557	76.1	490	23.9	2047

Caste/ethnicity					
Hill Brahmin	769	69.1	343	30.9	1112
Hill Chhetri	1141	63.6	654	36.4	1794
Terai Brahmin/Chhetri	125	75.1	41	24.9	166
Other Terai caste	1057	66.3	536	33.7	1593
Hill Dalit	476	60.8	308	39.2	784
Terai Dalit	301	62.7	179	37.3	481
Newar	362	80.1	90	19.9	452
Hill Janajati	1283	64.2	717	35.8	2000
Terai Janajati	674	70.5	282	29.5	956
Muslim	306	60.7	198	39.3	504
Others	27	83.8	5	16.2	32

Had an induced abortion in the last five years					
No	6199	66.0	3190	34.0	9389
Yes	322	66.3	164	33.7	486

Ever had emergency contraceptives					
No	6435	66.1	3299	33.9	9734
Yes	86	61.0	55	39.0	141
**Total**	**6522**	**66.0**	**3533**	**34.0**	**9875**

**Table 2:** Use of any method of FP (%) and its difference by cohabitation status among married women, 2016.

Women's characteristic	Cohabiting		Noncohabiting		Difference
	Any method	(*N*)	Any method	(*N*)	
Living children					
None	21.4	648	5.1	377	16.3
1–2	69.9	3066	18.8	1978	51.1
3–4	78.1	2148	40.3	817	37.8
5+	68.2	659	37.0	181	31.2

Age-groups (years)					
15–19	33.4	418	8.2	286	25.2
20–24	50.1	929	9.8	755	40.3
25–29	65.6	1118	19.5	839	46.1
30–34	76.2	1084	28.9	642	47.3
35–39	79.9	1075	40.4	436	39.5
40–44	77	1030	38.5	253	38.5
45–49	68.2	868	48.0	143	20.2

Highest educational level					
No education	69.5	2784	32.1	1200	37.4
Primary	66.3	1182	22.3	670	44
Secondary	64.5	1797	17.8	1094	46.7
Higher	69.9	758	15.0	389	54.9

Place of residence					
Urban	69.6	4112	23.1	1919	46.5
Rural	64.2	2409	24.0	1434	40.2

Province					
Province 1	72.9	1118	18.2	537	54.7
Province 2	59.8	1329	28.5	839	31.3
Province 3	70.7	1446	30.0	473	40.7
Province 4	72.1	553	15.6	397	56.5
Province 5	63.4	1150	18.4	599	45.0
Province 6	65.5	388	23.0	198	42.5
Province 7	73.5	537	29.2	309	44.3

Ecological zone					
Mountain	68.9	396	23.1	180	45.8
Hill	70.2	2791	20.5	1359	49.7
Terai	65.3	3334	25.8	1814	39.5

Wealth index					
Poorest	63.5	1107	21.6	580	41.9
Poorer	70	1235	24.6	711	45.4
Middle	66.3	1263	24.0	825	42.3
Richer	64.6	1359	23.8	749	40.8
Richest	72.3	1557	22.5	490	49.8

Ethnicity^*∗*^					
Hill Brahmin	76.6	769	17.7	343	58.9
Hill Chhetri	70.1	1141	19.0	654	51.1
Terai Brahmin/Chhetri	70.8	125	20.4	41	50.4
Other Terai caste	61	1057	30.8	536	30.2
Hill Dalit	69.2	476	22.5	308	46.7
Terai Dalit	51.5	301	26.4	179	25.1
Newar	67.6	362	32.1	90	35.5
Hill Janajati	69.7	1283	19.5	717	50.2
Terai Janajati	77.6	674	38.6	282	39
Muslim	39.2	306	14.8	198	24.4

Had an induced abortion in the last five years					
No	67.4	6199	24.0	3190	43.4
Yes	72.5	322	12.5	164	60

Ever had emergency contraceptives					
No	67.6	6435	23.5	3299	44.1
Yes	67.6	86	24.6	55	43

**Total**	**67.6**	**6521**	**23.5**	**2354**	44.1

^*∗*^Missing = 32.

**Table 3:** Unmet need for FP(%) and its difference by cohabitation status among married women in Nepal, 2016.

Women's characteristic	Cohabiting		Noncohabiting		Difference
	Any method	Total (*N*)	Any method	Total (*N*)	
Living children					
None	9.7	648	36.9	377	27.2
1–2	11.1	3066	56.3	1978	45.2
3–4	9.4	2148	41.5	817	32.1
5+	11.4	659	38.9	181	27.5

Age-groups (years)					
15–19	18.4	418	59.0	286	40.6
20–24	13.8	929	55.6	755	41.8
25–29	13.3	1118	52.4	839	39.1
30–34	9.0	1084	50.9	642	41.9
35–39	7.1	1075	41.8	436	34.7
40–44	7.6	1030	37.9	253	30.3
45–49	8.6	868	20.6	143	12.0

Highest educational level					
No education	9.4	2784	37.8	1200	28.4
Primary	11.1	1182	53.5	670	42.4
Secondary	12.4	1797	59.4	1094	47.0
Higher	8.7	758	51.5	389	42.8

Place of residence					
Urban	9.8	4112	50.4	1919	40.6
Rural	11.5	2409	48.5	1434	37.0

Province					
Province 1	9.4	1118	57.2	537	47.8
Province 2	10.2	1329	37.0	839	26.8
Province 3	9.9	1446	50.2	473	40.3
Province 4	9.2	553	59.0	397	49.8
Province 5	13.8	1150	54.9	599	41.1
Province 6	12.9	388	50.8	198	37.9
Province 7	7.0	537	46.1	309	39.1

Ecological zone					
Mountain	10.3	396	50.1	180	39.8
Hill	10.7	2791	56.1	1359	45.4
Terai	10.2	3334	44.7	1814	34.5

Wealth index					
Poorest	14.0	1107	51.8	580	37.8
Poorer	8.8	1235	49.8	711	41.0
Middle	9.3	1263	47.1	825	37.8
Richer	10.6	1359	47.7	749	37.1
Richest	9.9	1557	53.9	490	44.0

Ethnicity					
Hill Brahmin	7.2	769	55.6	343	48.4
Hill Chhetri	10.2	1141	56.3	654	46.1
Terai Brahmin/Chhetri	8.9	125	40.4	41	31.5
Other Terai caste	11.4	1057	36.6	536	25.2
Hill Dalit	15.3	476	56.7	308	41.4
Terai Dalit	13.3	301	35.3	179	22.0
Newar	11.8	362	52.7	90	40.9
Hill Janajati	10.2	1283	58.4	717	48.2
Terai Janajati	5.2	674	37.7	282	32.5
Muslim	17.3	306	40.9	198	23.6

Had an abortion in the last five years					
Yes	16.9	322	67.4	164	50.5
No	10.1	6199	48.7	3190	38.6

Ever had emergency contraceptives					
Yes	18.1	86	60.8	55	42.7
No	10.3	6435	49.4	3299	39.1
**Total**	**10.4**	**6521**	**49.6**	**2354**	**39.2**

^*∗*^Missing = 32.

**Table 4:** Factors associated with CPR (any method) and unmet need among cohabiting and noncohabiting women in Nepal, 2016.

Women's characteristic	CPR (any method)		Unmet need	
	Cohabiting	Noncohabiting	Cohabiting	Noncohabiting
	Adjusted odds ratio^*∗*^	aOR^*∗*^	aOR^*∗*^	aOR^*∗*^
Living children				
None	1	1	1	1
1–2	7.03 (5.59–8.84)	2.97 (1.74–5.06)	1.95 (1.35–2.81)	3.64 (2.73–4.85)
3–4	12.90 (9.89–16.82)	6.10 (3.41–10.91)	2.26 (1.41–3.63)	3.82 (2.73–5.34)
5+	9.66 (6.92–13.49)	4.25 (2.16–8.36)	2.82 (1.60–4.98)	5.39 (3.33–8.73)

Age-groups (years)				
15–19	1	1	1	1
20–24	0.97 (0.69–1.35)	1.01 (0.56–1.83)	0.60 (0.41–0.90)	0.50 (0.36–0.70)
25–29	1.24 (0.86–1.77)	1.93 (1.10–3.38)	0.53 (0.34–0.83)	0.35 (0.25–0.51)
30–34	1.83 (1.27–2.64)	3.18 (1.73–5.86)	0.32 (0.21–0.50)	0.29 (0.20–0.43)
35–39	2.21 (1.49–3.28)	5.64 (3.03–10.50)	0.25 (0.16–0.39)	0.21 (0.14–0.31)
40–44	1.84 (1.19–2.85)	5.50 (2.87–10.53)	0.26 (0.15–0.45)	0.15 (0.09–0.25)
45–49	1.00 (0.67–1.49)	7.30 (3.47–15.34)	0.31 (0.18–0.53)	0.07 (0.04–0.13)

Level of education				
No education	1	1	1	1
Primary	0.92 (0.76–1.12)	1.16 (0.86–1.57)	1.05 (0.81–1.38)	1.23 (0,95–1.59)
Secondary	1.06 (0.86–1.32)	1.43 (1.07–1.89)	1.26 (0.97–1.64)	1.32 (1.03–1.67)
Higher	1.44 (1.02–2.02)	1.45 (0.93–2.28)	0.91 (0.59–1.39)	0.97 (0.68–1.37)

Place of residence				
Urban	1	1	1	1
Rural	0.92 (0.77–1.08)	1.10 (0.87–1.40)	1.06 (0.85–1.31)	1.04 (0.87–1.25)

Province				
Province 1	1	1	1	1
Province 2	0.69 (0.50–0.96)	1.14 (0.69–1.91)	0.90 (0.59–1.38)	0.83 (0.57–1.21)
Province 3	0.76 (0.57–1.01)	2.51 (1.42–4.44)	1.07 (0.76–1.51)	0.65 (0.42–1.03)
Province 4	0.77 (0.58–1.01)	1.01 (0.62–1.63)	0.92 (0.62–1.36)	0.86 (0.60–1.22)
Province 5	0.63 (0.47–0.84)	0.92 (0.58–1.45)	1.49 (1.05–2.09)	0.97 (0.70–1.33)
Province 6	0.77 (0.58–1.03)	2.13 (1.25–3.61)	1.13 (0.76–1.68)	0.61 (0.41–0.91)
Province 7	0.81 (0.63–1.06)	2.12 (1.38–3.25)	0.77 (0.51–1.16)	0.58 (0.41–0.83)

Ecological zone				
Mountain	1	1	1	1
Hill	0.95 (0.70–1.30)	0.78 (0.44–1.37)	1.12 (0.75–1.67)	1.24 (0.77–2.00)
Terai	0.97 (0.67–1.39)	0.95 (0.48–1.88)	1.08 (0.67–1.74)	1.18 (0.70–2.00)

Wealth quintile				
Poorest	1	1	1	1
Poorer	1.66 (1.35–2.05)	1.20 (0.82–1.76)	0.61 (0.46–0.82)	1.15 (0.84–1.57)
Middle	1.73 (1.35–2.23)	1.09 (0.72–1.65)	0.63 (0.45–0.88)	1.18 (0.87–1.61)
Richer	1.54 (1.20–1.97)	1.06 (0.65–1.74)	0.69 (0.49–0.97)	1.12 (0.81–1.57)
Richest	1.91 (1.43–2.56)	0.98 (0.52–1.85)	0.71 (0.50–1.01)	1.41 (0.88–2.25)

Caste/ethnicity				
Hill Brahmin	1	1	1	1
Hill Chhetri	0.85 (0.64–1.14)	1.07 (0.69–1.66)	1.20 (0.82–1.75)	1.05 (0.75–1.46)
Terai Brahmin/Chhetri	0.77 (0.43–1.37)	1.57 (0.57–4.31)	1.35 (0.69–2.64)	0.46 (0.23–0.90)
Other Terai caste	0.52 (0.34–0.79)	3.17 (1.85–5.43)	1.54 (0.93–2.57)	0.33 (0.21–0.52)
Hill Dalit	0.96 (0.71–1.30)	1.59 (1.01–2.49)	1.79 (1.15–2.78)	0.98 (0.67–1.43)
Terai Dalit	0.41 (0.24–0.70)	2.12 (1.07–4.19)	1.95 (1.11–3.43)	0.37 (0.22–0.61)
Newar	0.73 (0.43–1.22)	2.61 (1.00–6.84)	1.87 (1.03–3.41)	0.75 (0.36–1.58)
Hill Janajati	0.94 (0.72–1.23)	1.45 (0.94–2.24)	1.21 (0.81–1.82)	1.00 (0.75–1.35)
Terai Janajati	1.27 (0.87–1.86)	4.32 (2.55–7.31)	0.69 (0.40–1.19)	0.39 (0.25–0.60)
Muslim	0.20 (0.13–0.31)	1.01 (0.50–2.02)	2.57 (1.45–4.53)	0.41 (0.25–0.67)

Had an abortion in the last five years				
No	1	1	1	1
Yes	1.08 (0.74–1.56)	0.42 (0.26–0.68)	1.62 (1.06–2.47)	1.65 (1.14–2.39)

Ever had emergency contraceptives				
No	1	1	1	1
Yes	0.79 (0.41–1.50)	1.59 (0.57–4.39)	2.02 (0.91–4.49)	1.28 (0.56–2.91)

^*∗*^All the variables presented are included in the model.

## Data Availability

The data used in this study are available at https://www.dhsprogram.com.

## References

[1] Ban B., Karki S., Shrestha A., Hodgins S. (2012). Spousal separation and interpretation of contraceptive use and unmet need in rural Nepal. *International Perspectives on Sexual and Reproductive Health*.

[2] Thapa N., Paudel M., Guragain A. M. (2019). *Status of Migration and Socio-Reproductive Impacts on Migrants and Their Families Left behind in Nepal*.

[3] Mehata S., Baral S., Chand P., Singh D., Poudel P., Barnett S. (2013). *Nepal Household Survey 2012*.

[4] Ministry of Health and Population (MoHP) Nepal, New ERA, and ICF International Inc (2017). *Nepal Demographic and Health Survey 2016*.

[5] Khanal M. N. (2013). *Impact of Male Migration on Contraceptive Use, Unmet Need, and Fertility in Nepal: Further Analysis of the 2011 Nepal Demographic and Health Survey*.

[6] Karandikar S., Coles L., Jayawant S., Kemp A. M. (2004). The neurodevelopmental outcome in infants who have sustained a subdural haemorrhage from non-accidental head injury. *Child Abuse Review*.

[7] Ministry of Labour and Employment Government of Nepal (2014). *Labour Migration for Employment, A Status Report for Nepal: 2013/14*.

[8] Gartaula H. N., Niehof A. (2013). Migration to and from the terai: shifting movements and motives. *The South Asianist*.

[9] Central Bureau of Statistics Nepal (2012). *National Population and Housing Census, 2011*.

[10] Clifford D. (2009). Spousal separation, selectivity and contextual effects: exploring the relationship between international labour migration and fertility in post-Soviet Tajikistan. *Demographic Research*.

[11] Lindstrom D. P., Giorguli Saucedo S. (2007). The interrelationship of fertility, family maintenance and Mexico-U.S. Migration. *Demographic Research*.

[12] Lindstrom D. P., Saucedo S. G. (2002). The short- and long-term effects of U.S. migration experience on Mexican women’s fertility. *Social Forces*.

[13] Agadjanian V., Yabiku S. T., Cau B. (2011). Men’s migration and women’s fertility in rural Mozambique. *Demography*.

[14] Millman S. R., Potter R. G. (1984). The fertility impact of spousal separation. *Studies in Family Planning*.

[15] Omondi D., Ochola C. (2003). Migration and fertility relationship: a case study of Kenya. *African Population Studies*.

[16] Jingyue Z. (2016). The influence of migration on Chinese Korean Women’s Fertility Behavior. *Studies in Asian Social Science*.

[17] Thapa S., Sharma S. K., Khatiwada N. (2014). Women’s knowledge of abortion law and availability of services in Nepal. *Journal of Biosocial Science*.

[18] Puri M., Singh S., Sundaram A., Hussain R., Tamang A., Crowell M. (2016). Abortion incidence and unintended pregnancy in Nepal. *International Perspectives on Sexual and Reproductive Health*.

[19] Ministry of Health Nepal (2019). *Annual Report, Department of Health Services 2074/2075 (2017/2018)*.

[20] Thapa S. (2016). A new wave in the quiet revolution in contraceptive use in Nepal: the rise of emergency contraception. *Reproductive Health*.

[21] Thapa S., Neupane S. (2013). Risk factors for repeat abortion in Nepal. *International Journal of Gynecology & Obstetrics*.

[22] Khan N. N. (2017). Seasonal migration induced spousal separation and infertility: growing evidences demand significance in developing countries. *International Journal of Perceptions in Public Health*.

[23] Khan R., MacQuarrie K., Nahar Q., Sultana M. (2016). The men are away: pregnancy risk and family planning needs among women with a migrant husband in Barisal, Bangladesh.

